# Effectiveness of a brief lay counsellor-delivered, problem-solving intervention for adolescent mental health problems in urban, low-income schools in India: a randomised controlled trial

**DOI:** 10.1016/S2352-4642(20)30173-5

**Published:** 2020-08

**Authors:** Daniel Michelson, Kanika Malik, Rachana Parikh, Helen A Weiss, Aoife M Doyle, Bhargav Bhat, Rooplata Sahu, Bhagwant Chilhate, Sonal Mathur, Madhuri Krishna, Rhea Sharma, Paulomi Sudhir, Michael King, Pim Cuijpers, Bruce Chorpita, Christopher G Fairburn, Vikram Patel

**Affiliations:** a School of Psychology, University of Sussex, Brighton, UK; b Sangath, Goa, India; c Sagath, New Delhi, India; d Department of Clinical Psychology, Vrije Universiteit, Amsterdam, Netherlands; e Medical Research Council Tropical Epidemiology Group, Faculty of Epidemiology and Population Health, London School of Hygiene & Tropical Medicine, London, UK; f Department of Clinical Psychology, National Institute of Mental Health and Neuro Sciences, Bengaluru, India; g Division of Psychiatry, Faculty of Brain Sciences, University College London, London, UK; h Department of Psychology, University of California, Los Angeles, CA, USA; i Department of Psychiatry, University of Oxford, Oxford, UK; j Department of Global Health and Social Medicine, Harvard Medical School, Boston, MA, USA; k Harvard TH Chan School of Public Health, Boston, MA, USA

## Abstract

**Background:**

Mental health problems are a leading cause of disability in adolescents worldwide. Problem solving is a well-tested mental health intervention in many populations. We aimed to investigate the effectiveness of a brief, transdiagnostic problem-solving intervention for common adolescent mental health problems when delivered by non-specialist school counsellors in New Delhi, India.

**Methods:**

This randomised trial was done in six government-run schools (three all-boys schools, two all-girls schools, and one co-educational school) that serve low-income communities. We recruited participants from grades 9 to 12 (ages 12–20 years) by selecting students with persistently elevated mental health symptoms accompanied by distress or functional impairment. Clinical eligibility criteria were assessed by research assistants using the Hindi-language version of the Strengths and Difficulties Questionnaire (SDQ), with reference to locally validated borderline cutoff scores of 19 or greater for boys and 20 or greater for girls on the SDQ Total Difficulties scale, an abnormal score of 2 or more on the SDQ Impact scale, and persistence of more than 1 month on the SDQ Chronicity index. Participants were randomly allocated (1:1) to problem solving delivered through a brief (2–3 week) counsellor-led intervention with supporting printed materials (intervention group), or problem solving delivered via printed booklets alone (control group). Primary outcomes were adolescent-reported mental health symptoms (SDQ Total Difficulties scale) and idiographic psychosocial problems (Youth Top Problems [YTP]) at 6 weeks. Primary analyses were done on an intention-to-treat basis at the 6-week endpoint. The trial is registered with ClinicalTrials.gov, NCT03630471.

**Findings:**

Participants were enrolled between Aug 20, and Dec 4, 2018. 283 eligible adolescents were referred to the trial, and 251 (89%) of these were enrolled (mean age 15·61 years; 174 [69%] boys). 125 participants were allocated to each group (after accounting for one participant in the intervention group who withdrew consent after randomisation). Primary outcome data were available for 245 (98%) participants. At 6 weeks, the mean YTP scores were 3·52 (SD 2·66) in the intervention group and 4·60 (2·75) in the control group (adjusted mean difference –1·01, 95% CI –1·63 to –0·38; adjusted effect size 0·36, 95% CI 0·11 to 0·61; p=0·0015). The mean SDQ Total Difficulties scores were 17·48 (5·45) in the intervention group and 18·33 (5·45) in the control group (–0·86, –2·14 to 0·41; 0·16, –0·09 to 0·41; p=0·18). We observed no adverse events.

**Interpretation:**

A brief lay counsellor-delivered problem-solving intervention combined with printed booklets seemed to have a modest effect on psychosocial outcomes among adolescents with diverse mental health problems compared with problem-solving booklets alone. This counsellor-delivered intervention might be a suitable first-line intervention in a stepped care approach, which is being evaluated in ongoing studies.

**Funding:**

Wellcome Trust.

## Introduction

Mental disorders account for around a sixth of the global burden of disease in adolescents (disability-adjusted life-years),^1^ and most forms of mental illness have their first onset before 18 years of age.^2^ Developmentally-appropriate mental health interventions are needed to prevent distress and disability at this crucial stage of life. Effective early interventions can also mitigate long-term risks for poor health, social exclusion, low economic activity, and other negative outcomes in adulthood.^3^ The scale and impact of adolescent mental disorders are especially pronounced in low-income and middle-income countries (LMICs), where healthy development is threatened by rapid social and economic change, increased urbanisation, the widening gap between rich and poor, youth unemployment, and gender disparities.^4^

The far-reaching implications of these challenges to youth mental health are evident in India, which is home to some 250 million 10 to 19-year-olds, 20% of the total adolescent population worldwide. High levels of stress are a feature of daily life for many adolescents,^5^ especially in metropolitan areas, as shown by the 2016 Indian National Mental Health Survey,^6^ which estimated that mental disorders are experienced by 13% of adolescents. India also has one of the highest youth suicide rates globally, and suicide is the leading cause of death in Indian adolescents.^7^

**Research in context**

**Evidence before this study**

Substantial research attests to the effectiveness of psychological interventions for adolescent mental health problems, but this evidence comes almost entirely from high-resource settings and high-intensity interventions delivered by mental health professionals. In previous studies involving adult populations in India, our group established the PRogramme for Effective Mental health Interventions in Under-resourced health systeMs (PREMIUM) methodology for developing evidence-based psychological treatments in culturally diverse, low-resource contexts. We extended the PREMIUM approach to a target population of school-going adolescents with common mental health problems, carrying out formative work in India over a period of 2·5 years (January, 2016 to June, 2018). Initial modelling, based on intervention design workshops with local and international experts in early 2016, supported a transdiagnostic, stepped care architecture focusing on anxiety, depression, and conduct difficulties. We additionally used a relevance mapping methodology to derive a list of candidate practice elements for inclusion in a suite of stepped interventions. Local data from epidemiological and help-seeking samples were cross-referenced with evidence from a database of over 1000 research trials on youth mental health interventions (PWEBS, via PracticeWise), which revealed what empirically-supported practice elements were most directly relevant to the demographic and mental health profiles of the reference samples. The findings were triangulated with qualitative data from 280 stakeholders, including adolescents, parents, school staff, and mental health practitioners. Problem solving was ultimately selected as the core component of a first-line intervention, on the grounds of parsimony, fit with local context, and potential for scalability.

**Added value of this study**

This study was designed to evaluate the first step in the PRemIum for aDolEscents (PRIDE) stepped care system and, to our knowledge, comprises one of the largest trials of a transdiagnostic adolescent mental health intervention in any country. We recruited a help-seeking sample of school-going adolescents with diverse mental health presentations, operationalised as scoring above cutoffs for overall symptom severity and impact (ie, distress and impairment). We found that a brief problem-solving intervention delivered by lay counsellors in four to five sessions over 3 weeks, combined with printed booklets, was superior to problem solving delivered via booklets alone in reducing idiographic problem severity, one of our two primary outcomes. We observed no moderation effects. The hypothesised mediator (perceived stress) accounted for 15% of the overall effect on idiographic problem severity. Effects on the primary outcome of mental health symptoms and secondary outcomes were more modest.

**Implications of all the available evidence**

A brief, lay counsellor-delivered problem-solving intervention helped to reduce self-reported psychosocial problem severity in adolescents with diverse mental health problems. However, the incremental benefit of the counsellor-delivered intervention compared with problem-solving booklets alone did not extend to effects on mental health symptom severity. Our results suggest that problem solving—delivered by counsellors where resources permit, or else by booklet—is a promising candidate as an initial brief intervention in a stepped care system for common adolescent mental health problems. A future study will evaluate the longer-term effects of the counsellor-delivered problem-solving intervention. Forthcoming research will also examine the effects of a complete stepped care intervention, in which more intensive psychological treatment is provided for adolescents who do not benefit from this brief, first-step intervention.

The negative impact of adolescent mental disorders is compounded by resource constraints—only 1·93 mental health workers are available in India per 100 000 population,^8^ with a tiny fraction of these dedicated to young people. Task sharing, involving existing or new cadres of workers with no previous training in mental health, is central to policy initiatives and practice innovations for improving mental health-care coverage in India and other LMICs.^9^ However, relatively few task-sharing interventions have been developed for adolescents affected by anxiety, depression, or conduct difficulties, which together account for 75% of the total adolescent mental health burden worldwide.^10^ Where available, evaluations of youth mental health interventions in LMICs have tended to focus on primary prevention for younger children and highly selective trauma-focused interventions in humanitarian contexts.^11^

The PRemIum for aDolEscents (PRIDE) research programme was implemented in India to address the scarcity of evidence-based interventions for common adolescent mental health problems nationally and in low-resource settings more widely. The goal was to develop and evaluate a suite of scalable, transdiagnostic psychological interventions (ie, suitable for various mental health presentations, such as anxiety, depression, and conduct difficulties) that could be delivered by non-specialist (so-called lay) counsellors in resource-poor school settings. This programme built upon India's national initiative for adolescent health, launched in 2014, which emphasised mental health as a public health priority and schools as an important platform for youth-focused treatment delivery.^12^

The development of the PRIDE school-based intervention model has been described in detail elsewhere.^13^ The model was founded on the principle of stepped care, which reserves increasingly specialised, resource-intensive interventions for individuals who do not respond to simpler first-line treatments.^14^ The intervention model was also influenced by key contextual findings, which included low mental health literacy and confidentiality concerns acting as barriers to help-seeking, adolescents' preference for practical advice rather than self-care, and the congested academic calendar that restricts the length and spacing of school-based intervention sessions.^15^ An intervention blueprint was produced through a series of iterative and recursive steps, and problem solving was ultimately selected as the core component of a low-intensity first-line intervention based on global evidence for its generalised transdiagnostic benefits across diverse mental health presentations and specific contextual relevance to coping with common stressors (eg, academic pressure and family conflict) observed in the target population. Intervention prototypes were subsequently refined in linked pilot studies.

The full PRIDE stepped care model includes an initial universal sensitisation component that uses both schoolwide and classroom-level activities to increase awareness about mental health problems and explain the purpose of counselling in clear and non-stigmatising terms, while offering explicit assurances about confidentiality. A lay counsellor-delivered brief problem-solving intervention (step 1) is then offered to adolescents with elevated mental health symptoms who refer themselves or are referred by teachers. A higher-intensity personalised psychological treatment (step 2) is offered to students who do not respond to the first-line problem-solving intervention.

We aimed to assess the first-line (step 1) intervention, specifically examining whether a lay counsellor-delivered problem-solving intervention (supported by printed booklets) was superior to a control condition (problem-solving booklets alone) in reducing the severity of adolescent-reported mental health symptoms and idiographic psychosocial problems at 6 weeks after randomisation. The full stepped care system and the specific effects of classroom sensitisation activities will be addressed in separate studies.

## Methods

### Study design and participants

This randomised trial was done in six government-run schools (three all-boys schools, two all-girls schools, and one co-educational school) that serve low-income communities in New Delhi, India. The study protocol has been previously published.^16^ Approvals were obtained from the Institutional Review Boards of Harvard Medical School, London School of Hygiene & Tropical Medicine, Sangath (the implementing institution in India), and the Indian Council of Medical Research.

Referrals were made by teachers or students themselves (via paper forms or in person), following a combination of whole-school and classroom-level sensitisation activities. The former involved briefings with school staff and posters advertising the availability of school counselling. Counsellors also visited individual classes (usually containing around 50 students at a time) to deliver a structured session that comprised an informational video about common mental health problems followed by a facilitated discussion about the counselling available in the trial. Research assistants accompanied counsellors on their classroom visits and were responsible for processing referrals, conducting subsequent eligibility and baseline assessments, and completing participant assent or consent procedures.

We recruited participants from grades 9 to 12 (ages 12–20 years) by selecting students with persistently elevated mental health symptoms accompanied by distress or functional impairment. Clinical eligibility criteria were assessed by research assistants using the Hindi-language version of the Strengths and Difficulties Questionnaire (SDQ),^17^ with reference to locally validated borderline cutoff scores of 19 or greater for boys and 20 or greater for girls on the SDQ Total Difficulties scale,^18^ an abnormal score of 2 or more on the SDQ Impact scale, and persistence of more than 1 month on the SDQ Chronicity index. Adolescents were excluded if they needed urgent medical attention, were receiving another mental health intervention, had taken part in previous PRIDE feasibility studies, or showed receptive or expressive language difficulties (written or spoken) that would affect their ability to participate fully in the trial procedures. Written assent (or consent for individuals aged 18 years or older) was obtained from all participating adolescents. Written consent was also sought from a parent or guardian (caregiver) for participation of adolescents younger than 18 years. A parallel consent process was used to obtain permission for use of caregiver-reported outcome measures. For quality assurance, we audiotaped assent or consent procedures with agreement from participants.

### Randomisation and masking

Participants were randomly assigned (1:1) to problem solving delivered through a brief (2–3 week) counsellor-led intervention with supporting printed materials (intervention group), or problem solving delivered via printed booklets alone (control group). An independent statistician generated a randomisation list using randomly sized blocks of four or six, stratified by school (and gender for the co-educational school). Allocations were concealed from field staff using sequentially numbered sealed envelopes. The envelopes were opened by a research assistant after each participant completed assent or consent procedures and baseline assessments. Follow-up assessments were done by a separate team of research assistants who were masked to allocation status. Allocation concealment was also applied to all coauthors (except the data manager, BB). Outcome assessments were completed by March 4, 2019, and primary analyses were completed before unmasking of the results on May 20, 2019.

### Procedures

Participants in the intervention group were offered training in problem solving on an individual basis, which consisted of four to five face to face sessions delivered over 2 to 3 weeks;^13^ the exact schedule was flexible depending on participant preference and availability. Each session lasted up to 30 min (ie, within the usual duration of school lessons), was delivered in Hindi, and recorded with permission. Students were excused from classes to attend sessions if they could not otherwise be arranged during free periods. Sessions took place on school premises in private rooms or, where private rooms were not available, behind screens and curtains that were erected for privacy in relatively quiet areas, such as libraries.

The first session introduced the principles of problem solving using the acronym POD, as follows: identify one or more current distressing or impairing problems (problem identification); identify ways of modifying the chosen problem or the accompanying emotional response, and select the most promising option (Option generation); and implement the chosen solution and evaluate the outcome (Do it). The next three sessions addressed each of the POD steps in greater depth and were supported by corresponding booklets. These A5-sized, full-colour booklets used contextually-appropriate comic strip stories to explain the steps of problem solving and illustrate how to cope with common difficulties. Home-based practice exercises were included in the booklets and discussed in the counselling sessions. The final session (number four or five) focused on consolidating what had been learned about problem solving and discussing future use in different situations.

Eight lay counsellors were deployed across the six schools, with each school being allocated one or two counsellors depending on demand. The counsellors were Hindi-speaking college graduates with no formal experience of delivering a psychological treatment. They were recruited through local advertisements and selected using a written aptitude test and interview.

The counsellors received a written manual describing the problem-solving intervention, followed by 5 days of office-based training, 6 weeks of supervised practice, and 3 top-up training days at monthly intervals while the trial was underway. Counsellors also participated in sensitisation activities aimed at promoting awareness and acceptability of counselling activities among school staff and pupils.^16^ These activities helped to integrate the counsellors within schools, although they were technically employed by the implementing non-governmental organisation (Sangath).

Counsellors typically spent 5 of 6 working days per week in schools, with 1 day per week assigned for office-based administrative tasks and supervision. The office-based administrative tasks and supervision comprised weekly peer group supervision meetings (lasting 2 h to 2·5 h), moderated by a rotating counsellor and overseen by a supervising masters-level or doctoral-level psychologist. In each meeting, the counsellors discussed one or two audio-recorded sessions that were rated by all group members using a therapy quality rating scale (appendix pp 8–11). Each counsellor also took part in a weekly 20 to 30 min telephone call with a supervisor to monitor the progress of their cases. The counsellors could also make ad hoc telephone calls if urgent consultation was needed with a supervisor because of any safeguarding concerns.

Participants in the control group received the same problem-solving booklets but without any additional counsellor input; the control was devised keeping in mind the requirement to offer a pragmatic, resource-efficient method of support with minimal risk of contamination between trial groups. The booklets were handed out immediately after random assignment by a research assistant (rather than a counsellor), who explained the purpose and content of the booklets according to a standardised script that lasted around 90 s. Participants were encouraged to read through the booklets in sequence and complete the specified practice exercises. No further guidance or support was provided.

### Outcomes

The primary outcomes, assessed using self-report measures administered at 6 weeks after randomisation, were severity of mental health symptoms, measured using the SDQ Total Difficulties score, and severity of self-defined psychosocial problems, measured using the Youth Top Problems (YTP)^19^ score.

The SDQ is the most widely used standardised measure of youth psychopathology globally and generates a total difficulties scale (scored from 0 to 40) made up of internalising and externalising subscales (each scored from 0 to 20), where higher scores indicate more severe symptoms. The YTP is a validated idiographic measure that identifies, prioritises, and scores the respondent's three main problems on a scale from 0 to 10, with a mean score obtained by averaging the individual problem ratings; higher scores indicate more severe problems. Both measures are routinely used in research and practice with diverse adolescent populations that span the target age range in the current study.

The rationale for having two primary outcomes was that problem solving would be directly relevant to managing an individual's prioritised problems, with concomitant effects on a global measure of mental health symptoms. Short-term outcomes were selected for the primary analyses in view of the planned stepped care context, in which participants would be expected to step up to a higher-intensity intervention if they did not respond to the problem-solving intervention.

The secondary outcomes (all adolescent-reported) were assessed during the 12 weeks after randomisation and were as follows: mental health symptoms (SDQ Total Difficulties score); idiographic problems (YTP score); distress and functional impairment (SDQ Impact score); peer relationship problems and emotional symptoms (SDQ Internalising symptoms subscale score); conduct problems and hyperactivity and inattention (SDQ Externalising symptoms subscale score); perceived stress (Perceived Stress Scale 4-item version [PSS-4^20^]); wellbeing (Short Warwick-Edinburgh Mental Wellbeing Scale^21^); and remission. Remission was defined using the crossing clinical threshold method^22^ applied to the SDQ Total Difficulties score (<19 for boys and <20 for girls) and any associated distress or functional impairment (SDQ Impact score <2).

Exploratory outcomes were also assessed over 12 weeks and were caregiver-reported SDQ scores (Total Difficulties, Impact, Internalising subscale, and Externalising subscale) and adolescent-reported SDQ Prosocial subscale score.

Existing validated Hindi-language translations were used for all outcome measures except the YTP, which was translated from English in consultation with a developer of the original measure (BC). Further information about the individual outcome measures and their administration, as well as descriptions of process data, are provided in the published trial protocol.^16^

### Statistical analysis

Assuming loss to follow-up of 15% over 6 weeks (based on piloting) and a 1:1 allocation ratio, we aimed to recruit at least 240 participants. This sample size would provide over 90% power to detect an effect size of 0·5 (and 80% power to detect an effect size of 0·4) at the α level of 0·025 for each of the two primary outcomes. We considered this a reasonably conservative estimate, given that effect sizes on both primary outcomes exceeded 0·9 in uncontrolled pilot studies.^13^

Baseline characteristics of enrolled participants (split by trial groups) and intervention process indicators were summarised by means and SDs, medians and IQRs, or frequencies and proportions. Trends in descriptive data were assessed using *t* tests or χ^2^ tests, as appropriate. For continuous outcomes, we plotted histograms for each group to assess normality and determine whether transformation was required. The results of outcome analyses were described in terms of strength of evidence of effect size and consistency of results for related outcomes rather than statistical significance. Therefore, we did not adjust p values for multiple comparisons.

Primary analyses were done on an intention-to-treat basis at the 6-week endpoint. These analyses were adjusted for baseline values of the outcome measure; school (as a fixed effect in the analysis), to allow for within-school clustering; counsellor variation (as a random effect); and variables for which randomisation did not achieve reasonable balance between the groups at baseline. For continuous outcomes with normally distributed errors (eg, SDQ Total Difficulties score), we estimated intervention effects using linear mixed-effects regression and reported them as adjusted mean differences and effect sizes, defined as standardised mean differences, with 95% CIs. For binary outcomes (eg, remission), we reported intervention effects as adjusted odds ratios (ORs) estimated from generalised (logistic) mixed-effects regression models with adjusted ORs and 95% CIs. We used repeated measures analysis to analyse outcomes for the two endpoints. Initial models included an interaction effect between group and time to allow for differential effects at 6 weeks and 12 weeks. This interaction effect was retained if there was evidence of effect modification by time (defined as p<0·05). We examined a dose–response effect in the intervention group by using mixed-effects regression models to assess differences in each of the primary outcomes according to frequency of session attendance. No interim analyses of outcomes were done.

We did exploratory moderation analyses to fit relevant interaction terms and tested for heterogeneity of intervention effects in regression models according to the following potential modifiers: baseline chronicity of mental health difficulties (≥12 months or >12 months based on SDQ Chronicity categories); baseline severity of mental health difficulties (borderline or abnormal SDQ scores); YTP typology (syndromic, functional, or both); and SDQ caseness profile (elevated Internalising symptoms subscale score, elevated Externalising symptoms subscale score, both subscale scores elevated, or neither subscale score elevated).

Additionally, mediation analysis was done to examine whether the theoretically derived a priori factor (perceived stress at 6 weeks) mediated the effects of the intervention on the severity of mental health symptoms and idiographic problems at 12 weeks. We controlled analyses for potential confounders, including baseline primary outcome and mediator scores according to the approaches used for the main trial analyses. We used generalised structural equation models with bootstrapped confidence intervals and the causal steps outlined by Baron and Kenny^23^ to examine associations between the intervention and the hypothesised mediator, the mediator and the outcomes, and the intervention and the outcomes.

Analyses were done with Stata version 15.1. A data safety and monitoring committee met at the outset of the trial and again at the time of unblinding the trial results. The trial is registered with ClinicalTrials.gov, NCT03630471.

### Role of the funding source

The funder of the study had no role in study design, data collection, data analysis, data interpretation, or writing of the report. The corresponding author had full access to all the data in the study and had final responsibility for the decision to submit for publication. The raw data were also accessible to the study's statisticians (HAW and AMD) and data manager (BB).

## Results

**Figure 1:**
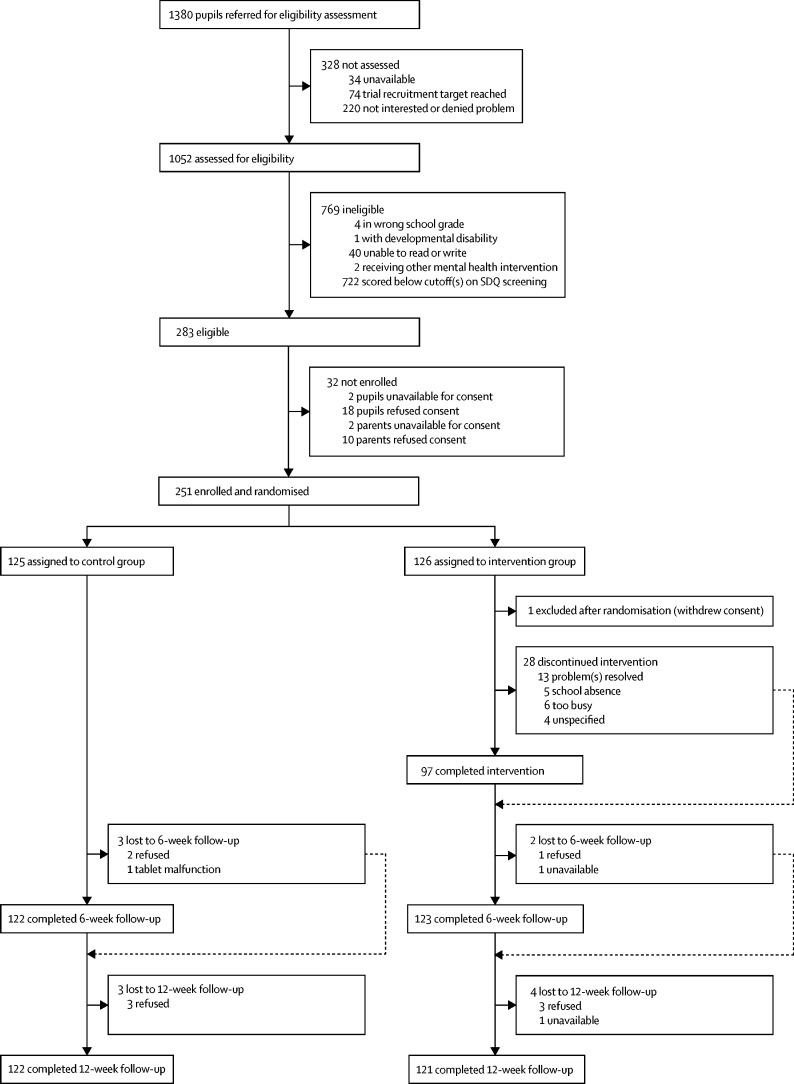
Trial profile SDQ=Strengths and Difficulties Questionnaire.

Participants were enrolled between Aug 20, and Dec 4, 2018 (figure 1). The total referred sample of 1380 adolescents comprised 781 (57%) students who had completed a self-referral form in their classroom after a sensitisation session, 411 (30%) so-called walk-ins who had self-referred by presenting directly at the school counsellor's room, and 138 (10%) adolescents who had self-referred by depositing their name in a box outside the school counsellor's room. 50 (4%) referrals were made by teachers. 251 (89%) of 283 eligible individuals were enrolled, all of whom were self-referrals by one of the methods. After accounting for one individual who declined consent retrospectively after random assignment, 125 participants were allocated to each group. Consenting participants were older than those who declined to participate (mean age 15·61 years [SD 1·68] *vs* 14·94 years [1·26]; p=0·028) but other demographic characteristics were similar (appendix p 1).

175 (70%) of 249 participants with baseline SDQ scores were in the abnormal range for psychopathology on the SDQ Total Difficulties scale, with the remainder being in the borderline range. SDQ subscale score profiles revealed abnormal case numbers of 133 (53%) for emotional symptoms, 103 (41%) for conduct problems, 115 (46%) for hyperactivity-inattention, and 107 (43%) for peer relationship problems, indicating that there was a high rate of coexisting difficulties and a roughly equal distribution of internalising and externalising problems within the sample. 134 (54%) of participants reported that their current difficulties had been present for more than a year.

Baseline demographic and clinical characteristics were similar across the two study groups and we did not adjust the analyses to account for baseline imbalances (table 1). Although we did not exclude siblings at recruitment, we did not identify any participants from the same household based on contact information provided. Outcome data were available on 245 (98%) participants at the 6-week endpoint, and 243 (97%) participants were assessed at the 12-week endpoint (figure 1). Participants with missing data at 6 weeks tended to be older but no other differences were discernible (appendix p 2).

**Table 1:** Baseline characteristics of the intention-to-treat population

		**Control group (n=125)**	**Intervention group (n=125)**	**Total (n=250)**
Sex				
	Females	38 (30%)	38 (30%)	76 (30%)
	Males	87 (70%)	87 (70%)	174 (70%)
Age, years		15·59 (1·68)	15·63 (1·68)	15·61 (1·68)
School grade				
	Ninth	52 (42%)	58 (46%)	110 (44%)
	Tenth	36 (29%)	31 (25%)	67 (27%)
	Eleventh	8 (6%)	8 (6%)	16 (6%)
	Twelfth	29 (23%)	28 (22%)	57 (23%)
Primary caregiver education*				
	No formal education	25/108 (23%)	25/111 (23%)	50/219 (23%)
	Completed primary school	3/108 (3%)	2/111 (2%)	5/219 (2%)
	Completed secondary school	56/108 (52%)	57/111 (51%)	113/219 (52%)
	Completed higher secondary and above	24/108 (22%)	27/111 (24%)	51/219 (23%)
Primary caregiver occupation†				
	Not employed outside the home	45/118 (38%)	46/118 (39%)	91/236 (39%)
	Manual	59/118 (50%)	52/118 (44%)	111/236 (47%)
	Office clerical	9/118 (8%)	5/118 (4%)	14/236 (6%)
	Professional	3/118 (3%)	4/118 (3%)	7/236 (3%)
	Other	2/118 (2%)	11/118 (9%)	13/236 (6%)
SDQ Total Difficulties score‡		23·12 (3·01)	23·22 (3·31)	23·17 (3·16)
SDQ Impact score‡		5·20 (2·37)	5·38 (2·41)	5·29 (2·38)
SDQ Internalising symptoms subscale score‡		11·96 (2·45)	12·09 (2·49)	12·02 (2·47)
SDQ Externalising symptoms subscale score‡		11·16 (2·40)	11·14 (2·37)	11·15 (2·38)
SDQ Chronicity‡				
	1–5 months	41/124 (33%)	35 (28%)	76/249 (31%)
	6–12 months	17/124 (14%)	22 (18%)	39/249 (16%)
	Over 12 months	66/124 (53%)	68 (54%)	134/249 (54%)
YTP score		7·35 (2·06)	7·24 (2·24)	7·30 (2·14)
PSS-4 score		9·22 (2·47)	9·04 (2·49)	9·13 (2·48)
SWEMWBS score		20·92 (5·28)	20·50 (4·82)	20·71 (5·05)

Data are n (%), mean (SD), or n/N (%). SDQ=Strengths and Difficulties Questionnaire. YTP=Youth Top Problems measure. PSS-4=Perceived Stress Scale 4-item version. SWEMWBS=Short Warwick-Edinburgh Mental Well-Being Scale.

* Primary caregiver refers to adult with joint or sole caring responsibility in the index adolescent's household. In the control group, this was based on 35 fathers, 61 mothers, eight brothers, and four sisters. In the intervention group, this was based on 30 fathers, 55 mothers, 16 brothers, and ten sisters.

† In the control group, this was based on 38 fathers, 68 mothers, eight brothers, and four sisters. In the intervention group, this was based on 30 fathers, 61 mothers, 16 brothers, and 11 sisters.

‡ Baseline SDQ was missing for one participant in the control group.

**Figure 2:**
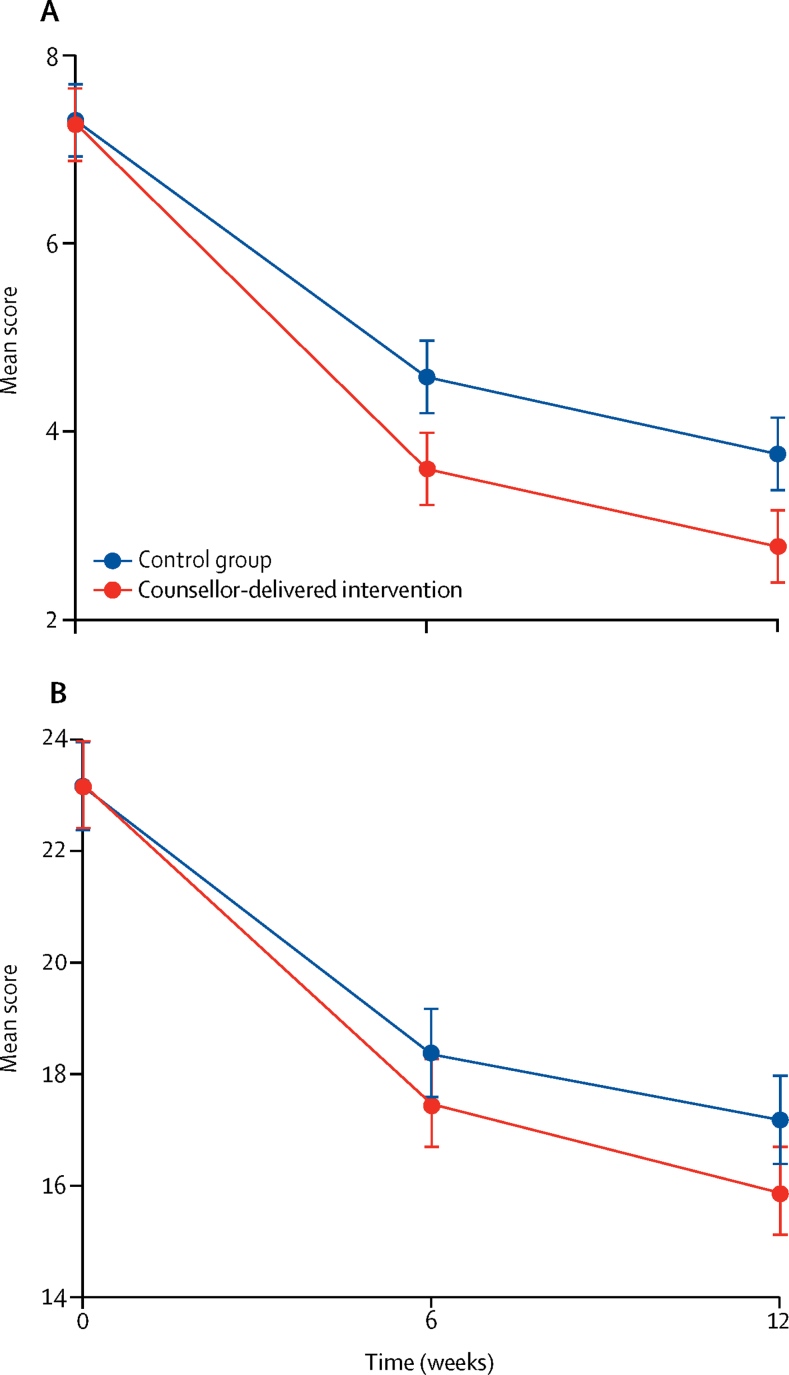
Primary outcomes over time according to study group (A) Mean Youth Top Problems measure score. (B) Mean Strengths and Difficulties Questionnaire Total Difficulties score. Error bars indicate 95% CIs.

We found an intervention group effect on YTP score at 6 weeks (adjusted mean difference –1·01, 95% CI –1·63 to –0·38; adjusted effect size 0·36, 95% CI 0·11 to 0·61; p=0·0015), but not on the SDQ Total Difficulties score (–0·86, –2·14 to 0·41; 0·16, –0·09 to 0·41; p=0·18; table 2). For both primary outcomes, we found no evidence of moderation by any of the candidate variables (appendix pp 3–4). The difference in mean YTP scores remained stable over 12 weeks (adjusted mean difference –1·03, 95% CI –1·60 to –0·47; adjusted effect size 0·35, 95% CI 0·18 to 0·54; p=0·0004; table 2; figure 2). Compared with YTP trajectories, SDQ Total Difficulties scores by group showed relatively greater divergence between 6 weeks and 12 weeks (adjusted mean difference –1·12, 95% CI –2·33 to 0·10; adjusted effect size 0·20, 95% CI 0·02 to 0·37; p=0·072; table 2; figure 2). The PSS-4 score at 6 weeks mediated 15% and 23% of the intervention group effects, respectively, on the YTP and SDQ Total Difficulties scores at 12 weeks (table 3).

**Table 2:** Primary and secondary outcomes

	**Control group**	**Intervention group**	**Adjusted mean difference or odds ratio (95% CI)**	**Adjusted effect size (95%CI)**	**p value**
**Primary outcomes (all adolescent-reported)**					
YTP score at 6 weeks	4·60 (2·75)	3·52 (2·66)	−1·01 (−1·63 to −0·38)	0·36 (0·11 to 0·61)	0·0015
SDQ Total Difficulties score at 6 weeks	18·33 (5·45)	17·48 (5·45)	−0·86 (−2·14 to 0·41)	0·16 (−0·09 to 0·41)	0·18
**Secondary outcomes (all adolescent-reported)**					
YTP score at 12 weeks	4·19 (2·89)	3·09 (2·69)	−1·03 (−1·60 to −0·47)	0·35 (0·18 to 0·54)	0·0004
SDQ Total Difficulties score at 12 weeks	17·70 (5·68)	16·69 (5·62)	−1·12 (−2·33 to 0·10)	0·20 (0·02 to 0·37)	0·072
SDQ Impact score at 12 weeks	1 (0 to 4)	0 (0 to 3)	−0·86 (−2·62 to 0·90)	0·23 (0·05 to 0·41)	0·34
SDQ Internalising symptoms subscale score at 12 weeks	8·73 (3·53)	8·21 (3·23)	−0·61 (−1·32 to 0·09)	0·18 (0·002 to 0·36)	0·089
SDQ Externalising symptoms subscale score at 12 weeks	8·97 (3·12)	8·48 (3·19)	−0·49 (−1·15 to 0·16)	0·16 (−0·02 to 0·33)	0·14
PSS-4 score at 12 weeks	7·17 (2·63)	6·62 (2·49)	−0·53 (−1·0 to −0·02)	0·21 (0·03 to 0·38)	0·040
SWEMWBS score at 12 weeks	23·27 (5·71)	24·18 (6·15)	1·00 (−0·27 to 2·28)	0·17 (−0·01 to 0·35)	0·12
Remission at 6 weeks	50/121 (41%)	50/123 (41%)	0·95 (0·56 to 1·64)	..	0·87
Remission at 12 weeks	58/121 (48%)	65/121 (54%)	1·31 (0·76 to 2·25)	..	0·33

Data are mean (SD), median (IQR), or n/N (%), unless otherwise indicated. SDQ=Strengths and Difficulties Questionnaire. YTP=Youth Top Problems measure. PSS-4=Perceived Stress Scale 4-item version. SWEMWBS=Short Warwick-Edinburgh Mental Well-Being Scale.

**Table 3:** Mediation effect of perceived stress measured at 6 weeks

	**Estimate**	**SE**	**p value**	**95% bootstrap CI**
**SDQ Total Difficulties score**				
Total effect: intervention effect on SDQ Total Difficulties score (12 weeks)	−1·24	0·73	0·088	−2·66 to 0·18
(a) Intervention effect on PSS-4 score (6 weeks)	−0·72	0·32	0·023	−1·34 to −0·10
(b) PSS-4 score (6 weeks) effect on SDQ Total Difficulties score (12 weeks)	0·38	0·16	0·016	0·07 to 0·70
Indirect effect: a × b*	−0·28	0·17	0·11	−0·62 to 0·06
**YTP score**				
Intervention effect on YTP score (12 weeks)	−1·00	0·32	0·0020	−1·64 to −0·37
(a) Intervention effect on PSS-4 score (6 weeks)	−0·72	0·32	0·023	−1·34 to −0·10
(b) PSS-4 score (6 weeks) effect on YTP score (12 weeks)	0·21	0·07	0·0020	0·08 to 0·34
Indirect effect: a × b*	−0·15	0·09	0·088	−0·32 to 0·02

SDQ=Strengths and Difficulties Questionnaire. PSS-4=Perceived Stress Scale 4-item version. YTP=Youth Top Problems measure.

* The proportion of the overall effect that is mediated by PSS-4 is 23% (−0·28/–1·24) for mean SDQ Total Difficulties score and 15% (−0·15/–1·00) for mean YTP score.

Scores on the continuous secondary outcomes decreased over 12 weeks in both groups (table 2). We found an effect on perceived stress that favoured the intervention group (adjusted effect size 0·21, 95% CI 0·03 to 0·38; p=0·040). SDQ Impact scores dropped substantially in both groups over 12 weeks, with a median of 0 (IQR 0 to 3) in the intervention group and 1 (0 to 4) in the control group, both registering below the abnormal cutoff score of 2. We found no evidence for an intervention group effect on remission (table 2). Our planned exploratory analyses found no evidence in favour of the intervention group (appendix p 5). No adverse events were reported in either group.

Process indicators for the intervention group are summarised in table 4. 97 (78%) of 125 participants in the intervention group completed the intervention (ie, attended four or more sessions). Reasons for non-completion were rapid resolution of the identified problems (13 [46%] of 28 participants), competing demands at school (six [21%] of 28 participants), persistent absence from school (five [18%] of 28 participants), and unspecified reasons (four [14%] of 28 participants; figure 1). Completers attended a mean of 4·83 sessions (SD 1·13) over 20·62 days (6·99) compared with 1·26 sessions (1·13) over 11·73 days (9·83) for non-completers (table 4). We found no association between the number of sessions attended and either primary outcome (appendix p 6).

**Table 4:** Intervention process indicators

		**Control group**	**Intervention group**
Sessions completed			
	Completers (n=97)*	..	4·83 (0·38) [4·76–4·91]
	Non-completers (n=27)	..	1·26 (1·13) [0·93–1·59]
Duration of intervention, days			
	Completers (n=97)	..	20·62 (6·99) [19·21–22·03]
	Non-completers (n=27)	..	11·73 (9·83) [6·99–16·47]
Duration of sessions, min		..	23·27 (4·31)
Participant adherence by session (counsellor-reported)			
	Sessions attended on time (across all sessions)	..	485/507 (96%)
	Sessions to which participants brought problem-solving booklets (sessions 2–4)	..	290/310 (94%)
	Sessions to which participants brought completed practice sheets (sessions 2–4)	..	297/310 (96%)
	Sessions in which participants reported implementation of problem-solving plan (sessions 4 and 5 only)	..	176/179 (98%)
Participant adherence at 6 weeks (adolescent-reported cumulative use of problem-solving booklets)			
	Never	9/121 (7%)	10/123 (8%)
	1–2 times	48/121 (40%)	27/123 (22%)
	3–4 times	31/121 (26%)	36/123 (29%)
	≥5 times	33/121 (27%)	50/123 (41%)
Participant adherence at 12 weeks (adolescent-reported cumulative use of problem-solving booklets)			
	Never	13/122 (11%)	9/121 (7%)
	1–2 times	29/122 (24%)	29/121 (24%)
	3–4 times	32/122 (26%)	22/121 (18%)
	≥5 times	48/122 (39%)	61/121 (50%)
Acceptability at 6 weeks (adolescent-reported perceived helpfulness of problem-solving booklets)			
	Not at all	12/121 (10%)	9/123 (7%)
	Slightly	61/121 (50%)	31/123 (25%)
	Very or extremely	48/121 (40%)	83/123 (67%)
Acceptability at 12 weeks (adolescent-reported perceived helpfulness of problem-solving booklets)			
	Not at all	10/122 (8%)	7/121 (6%)
	Slightly	49/122 (40%)	33/121 (27%)
	Very or extremely	63/122 (52%)	81/121 (67%)
Acceptability at 12 weeks (adolescent-reported service satisfaction: mean CSQ-8 total score)†		25·00 (4·46) [24·20–25·80]	26·55 (4·10) [25·81–27·29]

Data are n (SD) [95% CI], mean (SD), or n/N (%). CSQ-8=Client Satisfaction Questionnaire 8-item version.

* Defined as attendance at four or more sessions.

† n=122 control group; n=120 intervention group.

Considering all 507 attended sessions, mean session duration was 23·27 min (SD 4·31; table 4). Quality assessments of intervention group sessions indicated good to excellent intervention quality based on ratings from peers (n=35; mean 3·63 [0·29]; possible range 1–4, where 4 reflects the highest quality), a supervisor (n=35; 3·54 [0·33]), and an independent rater (n=49; 3·79 [0·29]).

More frequent use of problem-solving booklets was reported in the intervention group compared with the control group up to 6 weeks (p=0·021), but this difference was no longer significant at 12 weeks (p=0·25). Participants in the intervention group were more likely than control group participants to assign higher ratings of helpfulness at 6 weeks (p<0·0001); this difference by group was smaller at 12 weeks (p=0·052). Higher overall user satisfaction scores were also observed in the intervention group (p=0·0053).

## Discussion

This trial compared two relatively low-intensity ways to deliver a problem-solving intervention for adolescents with mental health symptoms in government-run schools that cater to low-income families in New Delhi, India. Problem solving delivered by lay school counsellors combined with booklets was effective in reducing the severity of adolescents' prioritised psychosocial problems compared with delivery using booklets alone. However, we found no evidence that the two formats differed on the outcome of mental health symptom severity, with similar reductions observed within both trial groups. Mean symptom scores at follow-up were close to the baseline eligibility threshold. We found no evidence of effect modification on either primary outcome, although the trial was not specifically powered to test for this. We found some evidence for the hypothesised mediator (perceived stress at 6 weeks), which is theoretically linked with problem-focused coping skills.^24^ We found no overall evidence of an effect of the counsellor-led problem-solving intervention on other outcomes, including adolescent-reported measures of wellbeing, severity of internalising and externalising symptoms, and impact scores.

The discrepant result for the two primary outcomes could be due to several reasons. First, the equivalence of the two delivery formats in terms of symptom reduction might be an indication that the control group constituted an active intervention. Research in high-income countries^25^ has found minimal effects of booklets on mental health outcomes in school settings, but it is possible that such formats are more potent in low-resource, low-income settings where other provision is negligible and expectancies might differ. Low uptake has been a common pitfall of other bibliotherapy approaches in high-income countries but we found evidence that problem-solving booklets were used in both groups of the trial; more frequent use was apparent in the intervention group up to 6 weeks but not over 12 weeks. A systematic review^26^ showed that self-help fared only slightly worse than face-to-face delivery for youth mental health problems and was equivalent when accompanied by therapist guidance or other interpersonal facilitation. Control group participants were able to participate in classroom sensitisation sessions led by a counsellor. Additionally, these participants had a brief introduction to the problem-solving booklets from a research assistant, and completed a series of outcome assessments that provided opportunities for reflecting on and sharing their problems confidentially. Any such relational enhancement of the booklets in the control group might have unintentionally minimised the putative incremental effects of the counsellor-led format in the intervention group. Second, the effects we observed were smaller than hypothesised and thus our trial was underpowered to reach statistical significance for the mental health symptoms outcome. By contrast, idiographic measures such as the YTP, which capture domains of personal importance to the respondent, are typically more responsive to psychotherapeutic change.^27^ Furthermore, person-centred idiographic measures are also more sensitive to within-person dynamic effects than are relatively global and stable nomothetic measures of psychopathology, which rely on group-based norms for validation and interpretation.^28^ This explanation could account for the finding that improvements in mental health symptoms lagged behind reductions in self-defined psychosocial problem severity. Symptom trajectories in our sample suggested that differences between the intervention and control groups were widening at 12 weeks, so it is possible that a longer follow-up period might have detected a delayed effect on mental health, perhaps as newly learned coping skills were practised and consolidated over time.

The absolute rates of clinical remission achieved by both groups were within the benchmarked range of 40–60% associated with evidence-based psychological treatments for adolescent mental health problems in high-income countries.^29^ The counsellor-led problem-solving format was incrementally more effective at improving an idiographic measure of adolescents' prioritised problems. Self-defined problems are likely to drive initial help-seeking and service retention, making this an important outcome in the target population. Therefore, we consider that problem solving—delivered by counsellors where resources permit, or at least by booklet if not—is a leading candidate as an initial brief intervention in a stepped care system for common adolescent mental health problems. Nonetheless, around half of participants in our study did not meet full remission criteria, which indicates the need for a more differentiated and intensive psychological treatment as a second sequential step (as in the design of the overall PRIDE programme). A key factor that underlies the usefulness of the problem-solving intervention is its brevity, permitting a rapid step-up for adolescents who do not show an adequate short-term response.

The need for scalable delivery formats is underscored by the high demand for counselling in this trial setting—nearly 16% of the student population in grades 9–12 were referred in the six schools over 3·5 months. Moreover, this number was accounted for almost entirely by self-referrals, which testifies to the value of engaging directly with adolescents with contextually-appropriate sensitisation activities, rather than relying on referrals from teachers or other adult gatekeepers. Nevertheless, almost three-quarters of referred students were deemed ineligible for the trial, primarily because they scored below clinical thresholds. Problem solving might have benefited this large group of adolescents who felt a need for psychological support but had subthreshold symptoms. Indeed, problem solving is the most common element in empirically supported adolescent mental health prevention programmes across the international literature, albeit with most evaluations originating from high-income settings.^30^ Low-intensity problem-solving interventions for indicated and universal prevention of adolescent mental health problems should be prioritised for further investigation in India and other LMICs.

Trial process indicators, which included follow-up rates, intervention adherence, and therapy quality scores, suggested high internal validity. Moreover, the findings are likely to be generalisable to routine settings, since the trial was implemented in government-run secondary schools that cater to the poorest urban communities in India. We also trained and deployed novice counsellors with a similar (or lesser) educational profile to the existing workforce of school counsellors in India. External validity was further strengthened through idiographic outcome assessment alongside standardised assessment instruments and relatively broad eligibility criteria that did not exclude any specific mental health presentations. Although there was an overrepresentation of boys among trial participants relative to the overall sampling frame, this can be explained in part by scheduling restrictions that were applied by staff in the all-girls schools during the trial's recruitment phase. Other potential sources of school-level variation (eg, school culture and leadership) were not specifically measured but could be an important focus for future process evaluations.

We acknowledge several study limitations. First, in the absence of a no-intervention control condition, we cannot rule out the contribution of spontaneous improvement as a reason for the outcome changes observed in the two trial groups. However, around half of participants reported that their problems had been present for longer than 12 months at the time of entering the trial. This relatively high level of chronicity mitigates against the likelihood of spontaneous remission over the relatively brief 12-week follow-up period. Second, the reported use of the booklets in both groups might reflect a reporting bias, considering the prevailing pressure in this school context for pupils to complete assigned homework, and potential concern about the consequences for not doing so. Third, diagnostic interviews were not done at baseline and we relied on other methods of phenotypic profiling within our clinically heterogeneous sample. However, the SDQ is the most widely used questionnaire for defining case-level morbidity in adolescent mental health trials globally and we used locally validated cutoffs. Finally, our follow-up period was relatively short, although this is in line with the conceptualisation of the intervention as the first step of a stepped care architecture. We intend to assess the sustainability of outcomes at 12-month follow-up in a future study, including an economic evaluation.

In conclusion, the current trial represents, to our knowledge, the largest evaluation of a school-based, lay counsellor-delivered intervention for common adolescent mental health problems. The trial is also one of the largest trials of a targeted, transdiagnostic mental health intervention for adolescents in any setting. Our findings show the value of a brief problem-solving intervention in reducing self-defined psychosocial problems among adolescents with diverse mental health presentations. Further research should focus on optimising the low-intensity delivery format, including the use of digital approaches that might reduce the costs associated with implementation by lay counsellors, and enhance acceptability over orthodox bibliotherapy formats. Future studies should also evaluate the delivery of problem solving within a more comprehensive and dynamic stepped care system, with the goal of improving overall remission from mental health problems. These intervention enhancements represent ongoing PRIDE studies.

## Data sharing

Anonymised participant data, data dictionary and case report forms will be made available by 12 months after trial completion. Data will be shared after approval by the corresponding author, following a reasonable submitted request. The study protocol and statistical analysis plan are publicly available.
