# Correction to *Lancet Child Adolesc Health* 2020; 4: 571–82

**DOI:** 10.1016/S2352-4642(20)30232-7

**Published:** 2020-09

*Michelson D, Malik K, Parikh R, et al. Effectiveness of a brief lay counsellor-delivered, problem-solving intervention for adolescent mental health problems in urban, low-income schools in India: a randomised controlled trial.* Lancet Child Adolesc Health *2020;* 4: *571–82*—In this Article, some of the secondary outcomes were incorrectly labelled in table 2. These corrections have been made to the online version as of July 23, 2020.

