# Supplementary material for: Effectiveness of a brief lay counsellor-delivered, problem-solving intervention for adolescent mental health problems in urban, low-income schools in India: a randomised controlled trial
Source: Lancet Child Adolesc Health. 2020 Aug;4(8):571–82. doi: 10.1016/S2352-4642(20)30173-5 (PMC7386943; doi:10.1016/S2352-4642(20)30173-5)
Supplement: Supplementary appendix [file mmc1.pdf]

THE LANCET
Child & Adolescent Health

Supplementary appendix

This appendix formed part of the original submission and has been peer reviewed. We post it as supplied by the authors.

Supplement to: Michelson D, Malik K, Parikh R, et al. Effectiveness of a brief lay counsellor-delivered, problem-solving intervention for adolescent mental health problems in urban, low-income schools in India: a randomised controlled trial. *Lancet Child Adolesc Health* 2020; published online June 22. http://dx.doi.org/10.1016/S2352-4642(20)30173-5.

Appendices

Table A1: Baseline characteristics of eligible adolescents who were enrolled and those who did not participate

	Consented (N=250)	Not consented (N=33)	p-value
**Gender (n [%])**			
Boys	174 (70%)	20 (61%)	0·30
Girls	76 (30%)	13 (39%)	
**Age in years (mean [SD])**	15·61 (1·68)	14·94 (1·26)	0·028
**School grade (n [%])**			
9th	110 (44%)	18 (55%)	0·45
10th	67 (27%)	8 (24%)	
11th	16 (6%)	3 (9%)	
12th	57 (23%)	4 (12%)	
**Baseline SDQ Total Difficulties Score (mean [SD])** [1]	23·17 (3·16)	23·03 (3.04)	0·81
**Baseline SDQ Impact score (mean [SD])** [1]	5·29 (2·38)	4·76 (2·29)	0·22
**Baseline SDQ Chronicity (n [%])**			
1-5 months	76 (30%)	9 (28%)	0·89
6-12 months	39 (16%)	6 (18%)	
More than 12 months	134 (54%)	18 (54%)	
Missing data	1 (0·4%)	0	

[1]Baseline SDQ missing for n=1 in consented group.

Table A2: Baseline characteristics of completers of outcome evaluation and participants lost to follow-up at 6-week end-point

	Lost before 6-week month follow-up (N=5)	Completed 6-week follow-up (N=245)	p-value
**Age (years) (mean [SD])**	17·08 (2·00)	15·58 (1·66)	0·05
**Gender (n [%])**			
Boys	3 (60%)	171 (70%)	0·64
Girls	2 (40%)	74 (30%)	
**School grade (n [%])**			
9th	1 (20%)	109 (45%)	0·41
10th	1 (20%)	66 (27%)	
11th	1 (20%)	15 (6%)	
12th	2 (40%)	55 (22%)	
**School (n [%])**			
GBSSS, Mahipalpur·	0 (0%)	63 (26%)	0·14
GBSSS, Badarpur·	0 (0%)	57 (23%)	
SBV, Badarpur·	2 (40%)	38 (16%)	
GGSSS, Badarpur·	2 (40%)	31 (13%)	
ASMS-SKV, Mahipalpur·	0 (0%)	32 (13%)	
SarvodayaV Co-Ed, Vasant Vihar	1 (20%)	24 (10%)	
**Week of enrolment (n [%])**			
2	0 (0%)	10 (4%)	0·74
3	0 (0%)	10 (4%)	
4	0 (0%)	18 (7%)	
5	1 (20%)	14 (6%)	
6	2 (40%)	44 (18%)	
7	0 (0%)	12 (5%)	
8	0 (0%)	28 (11%)	
9	2 (40%)	61 (25%)	
10	0 (0%)	47 (19%)	
11	0 (0%)	1 (0%)	
**SDQ Total Difficulties Score (mean [SD])**	23·00 (4·06)	23·18 (3·15)	0·90
**SDQ Impact score (mean [SD])**	5·60 (3·05)	5·29 (2·38)	0·77
**SDQ Internalising symptoms sub-scale score (mean [SD])**	12·60 (3·65)	12·01 (2·45)	0·60
**SDQ Externalising symptoms sub-scale score (mean [SD])**	10·40 (2·30)	11·16 (2·39)	0·48
**SDQ Prosocial sub-scale score (median [IQR])**	9·00 (7·00, 10·00)	8·00 (6·50,9·00)	0·47
**SDQ Chronicity (n [%])**			
1-5 months	1 (20%)	75 (31%)	0·87
6-12 months	1 (20%)	38 (16%)	
More than 12 months	3 (60%)	131 (54%)	
**YTP score (median [IQR])**	8·00 (8·00,10·00)	7·67 (6·00, 9·00)	0·33
**PSS-4 score (mean [SD])**	9·80 (0·84)	9·11 (2·50)	0·54
**SWEMWBS score (mean [SD])**	18·40 (3·91)	20·76 (5·07)	0·30

Table A3: SDQ Total Difficulties score by effect modifiers: adjusted SDQ Total Difficulties scores at 6 weeks

	N	Control: Booklets only (mean [SD])	Intervention: Counsellor-delivered problem-solving intervention plus booklets (mean [SD])	Intervention effect: adjusted mean difference[1] (95% CI); p-value
**Baseline chronicity of mental health difficulties**				
<=12 months	115	17·67 (5·33)	17·36 (5·54)	-0·35 (-2·26,1·55); p=0·72
>12 months	134	18·91 (5·58)	17·58 [5·41)	-1·31 (-3·06, 0·45); p=0·15
				p-value for effect-modification=0·48
**Baseline severity of mental health difficulties**				
Borderline	74	16·75 (4.87)	15.50 (5·27)	-1.14 (-3.54, 1·25); p=0·35
Abnormal	175	18.89 (5·59)	18·43 (5·31)	-0.77 (-2·30, 0·76); p=0·33
				p-value for effect-modification=0·80
**YTP type**				
Syndromic	40	19·00 (4·20)	17·55 (7·18)	-1·19 (-4·46, 2·07); p=0·47
Functional	76	17·11 (5·13)	17·00 (4·67)	-0·81 (-3·14, 1·53); p=0·50
Both	134	18·85 (5·85)	17·73 (5·27)	-0·80 (-2·56, 0·96); p=0·37
				p-value for effect-modification=0·98
**SDQ caseness profile**				
Elevated Internalising symptoms sub-scale	64	18·87 (5·43)	16·09 (4·91)	-2·34 (-4·85, 0·17); p=0·07
Elevated Externalising symptoms sub-scale	52	16·00 (6·47)	16·64 (6·53)	0·63 (-2·16, 3·41); p=0·66
Both sub-scales elevated	121	19·10 (4·93)	19·10 (4·83)	-0·38 (-2·20, 1·44); p=0·69
Neither sub-scale elevated	12	17·50 (3·70)	13·14 (4·81)	-4·23 (-10·52, 2·05); p=0·19
				p-value for effect-modification=0·29

[1]Adjusted for baseline value of outcome, age group, school and counsellor.

Table A4: YTP mean score by effect modifiers: adjusted mean YTP scores at 6 weeks

	N	Control: Booklets only (mean [SD])	Intervention: Counsellor-delivered problem-solving intervention plus booklets (mean [SD])	Intervention effect: adjusted mean difference[1] (95% CI); p-value
**Baseline chronicity of mental health difficulties**				
<=12 months	115	4·28 (2·50)	3·40 (2·57)	-0·78 (-1·72, 0·15); p=0·10
>12 months	134	4·96 (2·90)	3·61 (2·74)	-1·24 (-2·10, -0·39); p=0·004
				p-value for effect-modification=0·48
**Baseline severity of mental health difficulties**				
Borderline	74	4·31 (2·59)	2.77 (2·43)	-1·19 (-2·35, -0·022); p=0·046
Abnormal	175	4·76 (2·78)	3·88 (2·70)	-0·90 (-1·64, -0·17); p=0·016
				p-value for effect-modification=0·69
**YTP type**				
Syndromic	40	4·27 (2·54)	2·75 (2·12)	-1·19 (-2·80, 0·41); p=0·15
Functional	76	3·93 (3·10)	3·86 (3·12)	-0·49(-1·64, 0·65); p=0·40
Both	134	5·06 (2·53)	3·58 (2·52)	-1·21 (-2·07, -0·35); p=0·006
				p-value for effect-modification=0·60
**SDQ caseness profile**				
Elevated Internalising sub-scale	64	4·26 (2·59)	2·74 (2·35)	-1·18 (-2·40, 0·04); p=0·06
Elevated Externalising sub-scale	52	3·62 (2·77)	3·09 (2·52)	-0·88 (-2·23, 0·48); p=0·21
Both sub-scales elevated	121	5·18 (2·69)	4·21 (2·85)	-0·88 (-1·76, -0·001); p=0·05
Neither sub-scale elevated	12	6·13 (2·22)	2·69 (1·42)	-2·73 (-5·79, 0·33); p=0·08
				p-value for effect-modification=0·71

[1]Adjusted for baseline value of outcome, age group, school and counsellor.

Table A5: Exploratory outcomes: intervention effects over 12 weeks

	At 6 weeks		At 12 weeks		Over 12 weeks				
**Outcome**	**Control: Booklets only**	**Intervention: Counsellor-delivered problem-solving intervention plus booklets**	**Control: Booklets only**	**Intervention: Counsellor-delivered problem-solving intervention plus booklets**	**Control**	**Intervention**	**Adjusted mean difference or prevalence ratio (95% CI)**	**Adj. effect size[1] (95% CI)**	**p-value**
**Caregiver-reported SDQ Total Difficulties score over 12 weeks (mean [SD])**	13·96 (5·17)	13·93 (5·78)	13·06 (5·35)	13·50 (5·90)	13·52 (5·27)	13·71 (5·83)	0·003 (-1·95, 1·96)	0·02 (-0·16, 0·21)	1.0
**Caregiver-reported SDQ Impact score over 12 weeks (median [IQR])**	0·00 (0·00, 0·00)	0·00 (0·00, 0·00)	0·00 (0·00, 0·00)	0·00 (0·00, 0·00)	0·00 (0·00, 0·00)	0·00 (0·00, 0·00)	-0·04 (-0·31, 0·23)	0·03 (-0·16, 0·21)	0·79
**Caregiver-reported SDQ Internalising sub-scale score over 12 weeks (mean [SD])**	6·96 (3·33)	6·67 (3·52)	6·46 (3·27)	6·28 (3·42)	6·71 (3·30)	6·47 (3·47)	-0·28 (-0·93, 0·38)	0·08 (-0·11, 0·27)	0·41
**Caregiver-reported SDQ Externalising sub-scale score over 12 weeks (mean [SD])**	7·01 (3·09)	7·26 (3·29)	6·60 (3·04)	7·22 (3·56)	6·81 (3·06)	7·24 (3·42)	0·25 (-1·25, 1·74)	0·13 (-0·06, 0·32)	0·74
**Adolescent-reported SDQ Prosocial sub-scale score over 12 weeks (median [IQR])**	7·00 (6·00, 9·00)	8·00 (6·00, 9·00)	8·00 (6·00, 9·00)	7·00 (6·00, 9·00)	7·50 (6·00, 9·00)	7·00 (6·00, 9·00)	-0·03 (-0·47, 0·41)	0·01 (-0·16, 0·19)	0·89

[1]Assumes equal standard deviation per arm.

Table A6: Dose-response analysis

	SDQ total score at baseline	YTP mean score at baseline	SDQ total score at 6 weeks[1, 2]	YTP mean score at 6 weeks[1, 2]
**Intervention compliance**				
Non-compliers (attended 0-3 sessions) (n=27, 22%)	23·07 (3·99)	7·09 (2·51)	17·31 (6·40)	3·85 (2·87)
Compliers (attended 4-5 sessions) (n=98, 78%)	23·27 (3·12)	7·28 (2·17)	17·53 (5·21)	3·43 (2·61)
			Coeff=0·04 (-2·27, 2·35), p=0·97	Coeff=-0·10 (-1·25, 1·04), p=0·86

Data are mean (SD) or n/N (%). [1]SDQ Total Difficulties and YTP scores at six weeks available for n=123. [2]Coefficients from mixed-effects regression model adjusting for baseline value of outcome, age group, school and counsellor.

Table A7: Response frequencies for service satisfaction measure items by arm

	Intervention arm (N=120)				Control arm (N=122)			
	1 (poor, no-definitely not, none of my needs met, quite dissatisfied, or made things worse)	2 (fair, no-not really, few needs met, mildly dissatisfied, really didn't help, or no-I don't think so)	3 (good, yes-generally, mostly satisfied, yes-helped somewhat, or yes-I think so)	4 (excellent, yes-definitely, almost all needs met, very satisfied, or helped a great deal)	1 (poor, no-definitely not, none of my needs met, quite dissatisfied, or made things worse)	2 (fair, no-not really, few needs met, mildly dissatisfied, really didn't help, or no-I don't think so)	3 (good, yes-generally, mostly satisfied, yes-helped somewhat, or yes-I think so)	4 (excellent, yes-definitely, almost all needs met, very satisfied, or helped a great deal)
**Rate the quality of the service you received ([n], %)**	1 (1%)	16 (13%)	47 (39%)	56 (47%)	3 (3%)	24 (20%)	58 (48%)	37 (30%)
**Did you get the kind of service you wanted? ([n], %)**	5 (4%)	10 (8%)	58 (48%)	47 (39%)	8 (7%)	17 (14%)	57 (47%)	40 (33%)
**Did the service meet your needs? ([n], %)**	4 (3%)	26 (22%)	48 (40%)	42 (35%)	14 (11%)	36 (30%)	41 (34%)	31 (25%)
**Would you recommend the service to a friend? ([n], %)**	4 (3%)	5 (4%)	24 (20%)	87 (73%)	2 (2%)	5 (4%)	30 (25%)	85 (70%)
**How satisfied are you with the amount of help you received? ([n], %)**	8 (7%)	25 (21%)	28 (23%)	59 (49%)	8 (7%)	25 (20%)	44 (36%)	45 (37%)
**Has the service helped you to deal more effectively with your problems? ([n], %)**	1 (1%)	3 (3%)	56 (47%)	60 (50%)	3 (3%)	10 (8%)	63 (52%)	46 (38%)
**Overall, how satisfied are you with the service you received? ([n], %)**	6 (5%)	17 (14%)	44 (37%)	53 (44%)	12 (10%)	29 (24%)	44 (36%)	37 (30%)
**If you were to seek help again, would you come back to the service? ([n], %)**	2 (2%)	8 (7%)	36 (30%)	74 (62%)	8 (7%)	15 (12%)	21 (17%)	78 (64%)

Rating and feedback form

PRIDE Therapy Quality Rating Scale (P-TQRS)- Step 1

**Session details**	Counsellor's name/ID:	Trial id	Session no	Date of session	Site: Delhi/ Goa
**Raters details**	Rater's name/ ID	Date of rating			
**Type of rating**	❑ Self-rating	❑ Peer rating	❑ Supervisor rating	❑ Fidelity rating	
**Mode of session**	❑ Audio tape	❑ Live session	❑ Written Transcript		
**Setting of Supervision**	❑ Individual session	❑ Group session			

Numerical Rating:

For each of the item on the scale, there is list of features provided in the corresponding column that describes what ideally the therapist need to cover in that domain during the session. Based on the features covered, each item is rated on 5-point scales, using the scoring scale given below:

Scoring Legend for Performance band*:

1 = **Limited**; skill not performed or inappropriate performance with major problems evident; skill delivery is not useful in session; majority of outlined features are missing
2 = **Basic**; skill performed is somewhat appropriate, some of the outlined features are covered, however there are substantive problems and/or inconsistencies in counsellor's performance;
3 = **Good**; Skill performed appropriately; most of the outlined features covered in systematic manner; however minimal problems and/or consistencies are evident in counsellor's performance
4 = **Advanced**; Skill is highly developed; consistently well-performed; all outlined features covered in systematic manner, suitable to student presenting problem and stage of counselling

*Not applicable is marked if the particular skill is not relevant for the particular session. This is to be used only for treatment specific skills.

Qualitative feedback:

After completing numerical ratings for each domain, assessors have been given space to write additional comments for each domain: strengths and learning needed. Assessor can draw on the features outlined for each domain to provide examples of what made some aspects of the counsellor's performance more successful than others. Where areas of weakness are identified, assessors can suggest alternative, more skillful ways of working within this domain and highlight ways in which the counsellor could further develop their skills in this area. In the end, the assessor can highlight overall areas of strength and learning needs. Such in-depth feedback is invaluable in aiding further development and progression.

PRIDE
LET THE SUN SHINE OVER YOU

S.no	Item	Description	Performance band	Comment
1.	Agenda setting	• Establishes a well-defined and specific agenda at start of the session, suited to stage of counselling and student's problems<br>• Establishes agenda that ensures all items could be thoroughly covered within the session duration<br>• Checks with students if its relevant for them<br>• Refers to treatment ladder to explain progress though treatment	1 2 3 4 NA	
2.	**Assessing risk and therapeutic change**			
a.	Progress monitoring (mood & problem rating scales)<br>*(Session 2 onwards)*	• Implements rating scales in systematic order and explains why these are done on a regular basis<br>• Reviews completed rating scales and uses information for relevant clinical purposes<br>• Explores barriers in understanding and using rating scales	1 2 3 4 NA	
b.	Monitoring risk for harm	• Conducts safety check and explains why these are done on a regular basis<br>• Allows the student adequate time to share his/her experience<br>• Skillfully and sensitively probes to establish appropriate level of risk<br>• Follows risk management protocol step by step in case of a moderate/high risk	1 2 3 4 NA	
**3.**	Formulation<br>*(Preferably during Session 2 & 5)*	• Helps student prioritize the target problem for counselling<br>• Shares with student a personalized formulation connecting problems and efforts at coping<br>• Uses clear and developmentally appropriate language<br>• Uses formulation sheet to share the formulation with student	1 2 3 4 NA	
4.	**Use of problem solving steps: problem identification, option generation, action planning and review**			
a.	Rationale for intervention	• Provides clear rationale for using problem solving intervention or for the particular step of problem solving being covered in the session<br>• Explain about concepts using clear and developmentally appropriate concepts<br>• If there were any ambiguities or doubts expressed by student, addressed them fully	1 2 3 4 NA	
b.	Implementation of intervention	• Implements the steps of problem solving interventions from start to finish in the session, in the manner in which they were intended<br>• Able to tailor interventions to suit the student's needs and presentation	1 2 3 4 NA	
**5.**	**To do List**			
**a.**	Reviews To do list<br>*(Session 2 onwards)*	• Reviews assigned To do list with the student (reading of POD resource material, completion of the worksheets and/or implementation of tasks given in POD booklets in real world)<br>• Help student understand the implications from completing/not completing To do list and to link this learning to their problems<br>• If required, counsellor worked with the student to identify reasons for non-completion and helped them identify specific ways to overcome future blocks to completion	1 2 3 4 NA	
**b.**	Rationale for To do list	• Introduced the materials and tasks that are part of To do list<br>• Skillfully facilitated the student's understanding of the purpose of To do list	1 2 3 4 NA	

**c.**	Choosing & Planning suitable To do list	• To do list (choice of POD resource material and implementations task) built upon important issues dealt with in-session or previous To do list<br>• Formulates a clear and detailed plan of exactly what this involve<br>• Prepares the student well for practicalities of To do list (how, when, where)<br>• Identifies any potential obstacles, fully discussed these obstacles and (where possible) identified ways to overcome them	1 2 3 4 NA	
**6.**	**Fostering Therapeutic relationship**			
**a.**	Engagement	• Demonstrated a positive interpersonal style<br>• Adapted their interpersonal style so that it was congruent with the student's presentation and stage of counselling	1 2 3 4 NA	
**b.**	Confidentiality *(Preferably during Session 1, repeated if required)*	• Explains that all discussions in counselling are confidential<br>• Explain about exception to confidentiality i.e. harm to self and others<br>• Explain the student purpose of audio-recording<br>• If the student did not understand, or asked questions, fully discussed the issues	1 2 3 4 NA	
**c.**	Collaboration	• Encouraged the student to take an active role in and to share responsibility for all aspects of the session<br>• Done in a manner that is suited to the stage of counselling and student's problem severity	1 2 3 4 NA	
**7.**	**Effective two-way communication**			
**a.**	Summaries	• At appropriately regular intervals within session as well as at end of session, elicits student's summaries of session content<br>• Provides clear, brief and meaningful summaries at regular interval as well as at end of session<br>• Discussed and appropriately responded to summaries	1 2 3 4 NA	
**b.**	Questioning and Feedback	• Used appropriate open ended questions to elicit feedback<br>• Checked the student's understanding of and reaction to session components<br>• Thoroughly and openly discussed student's feedback and responded appropriately	1 2 3 4 NA	
**8.**	Effective use of time	• Maintained focus on session priorities (i.e. agenda items).<br>• Ability to pace the session in a manner which is well suited to agenda and student's capacity<br>• If unanticipated issues arose, counsellor acknowledged these, skillfully evaluated and showed appropriate flexibility	1 2 3 4 NA	

Score: _____________________

Mean score: Score/(15-NA's):_________________

How complex do you think the case was to work with?

- ☐ Very straightforward
- ☐ Somewhat straightforward
- ☐ Somewhat complex
- ☐ Very complex

Provide explanation for ratings:____

Skills profile

Counsellor's Key Strengths

What were the counsellor's key strengths during the session?

Counsellor's Learning Needs

Based on the session assessed, what skills does the counsellor need to concentrate on improving further? How could he/she develop skills in those areas?
